# The Challenge of Producing Ubiquitinated Proteins for Structural Studies

**DOI:** 10.3390/cells3020639

**Published:** 2014-06-12

**Authors:** Serena Faggiano, Annalisa Pastore

**Affiliations:** 1 National Institute for Medical Research, MRC, The Ridgeway, NW71AA London, UK; E-Mail: sfaggia@nimr.mrc.ac.uk; 2 Department of Clinical Neuroscience, King’s College London, Denmark Hill Campus, SE58AF London, UK

**Keywords:** ubiquitin, post-translational modification, mono-ubiquitination, isopeptide bond, native chemical ligation, non-enzymatic ubiquitination, enzymatic ubiquitination, *in vitro* ubiquitination, X-ray crystallography, nuclear magnetic resonance spectroscopy

## Abstract

Protein ubiquitination is an important post-translational modification involved in several essential signalling pathways. It has different effects on the target protein substrate, *i.e.*, it can trigger the degradation of the protein in the proteasome, change the interactions of the modified protein with its partners, or affect its localization and activity. In order to understand the molecular mechanisms underlying the consequences of protein ubiquitination, scientists have to face the challenging task of producing ubiquitinated proteins for structural characterization with X-ray crystallography and/or nuclear magnetic resonance (NMR) spectroscopy. These techniques require milligrams of homogeneous samples of high purity. The strategies proposed so far for the production of ubiquitinated proteins can be divided into two groups, *i.e*., chemical (or non-enzymatic) and enzymatic methodologies. In this review, we summarize the still very sparse examples available in the literature that describe successful production of ubiquitinated proteins amenable for biochemical and structural studies, and discuss advantages and disadvantages of the techniques proposed. We also give a perspective of the direction in which the field might evolve.

## 1. Introduction

Protein ubiquitination consists of the covalent attachment of the ε-amino group of a target protein lysine to the carboxylic group of the ubiquitin (Ub) C-terminal glycine via an isopeptide bond. Proteins can be mono-, multi-, or poly-ubiquitinated. Ub can, in fact, cross-link itself to another Ub by modification of the N-terminus and attachment to any of the seven lysine residues of another Ub, which leads to the formation of polymeric chains. All possible linkages of the seven lysines of Ub, including mixed ones, have been observed *in vivo*. Each linkage is thought to be associated with a specific function, suggesting that ubiquitination acts as a “protein code”, able to store and transmit information; ubiquitination not only targets proteins for degradation in the proteasome, but also alters protein localization, activity and interaction with binding partners, depending on the linkage [1,2,3]. The length of these chains can be confined to only two Ub molecules or be as long as ten or more moieties. *In vivo*, Ub conjugation is performed by a cascade of events involving three classes of enzymes called Ub-activating enzymes (E1), Ub-conjugating enzymes (E2) and Ub ligases (E3), and is reversed by deubiquitinating enzymes (DUBs).

The molecular mechanisms responsible for the effects of non-proteolytic protein ubiquitination are in most cases elusive, but seem to be important in several completely different pathways. Examples of proteins that undergo this post-translational modification include mono-ubiquitination of the proliferating cell nuclear antigen (PCNA) and the multiple mono-ubiquitination of the epidermal growth factor receptor (EGFR). Many proteins that undergo ubiquitination are also involved in the development of human diseases, such as the transcription factor p53 and the oncogene Ras. Also E2, E3 and DUBs, novel potential targets for anti-cancer drug discovery, are modified by Ub or Ub-like proteins in cells. This strongly supports the importance of studying how ubiquitination modifies the structure and function of proteins and the role of poly-Ub chains of different linkages.

Biochemical, biophysical and structural studies are essential to shed light on these crucial regulatory pathways. These studies, however, require variable but significant amounts of highly pure protein, ranging from micrograms to milligrams, depending on the technique chosen. For example, few micrograms of a mono-ubiquitinated sample are in general sufficient for the characterization of the changes that occur in the secondary structure of a protein upon ubiquitination using circular dichroism (CD). On the other hand, measuring the changes of the binding affinity to protein partners upon ubiquitination by isothermal calorimetry (ITC) requires larger quantities of ubiquitinated protein. Structural biology techniques, such as X-ray crystallography and nuclear magnetic resonance (NMR), also demand the production of several milligrams of samples. The homogeneity of the sample is also a crucial prerequisite for structural characterization.

Protein ubiquitination assays are routinely performed in biochemical studies by enzymatic ligation and subsequent SDS-PAGE or Western blot analysis (*in vitro* enzymatic ubiquitination), if the specific E2 and E3 enzymes are known. However, a biochemical and structural characterization of a ubiquitinated protein requires large amounts of a homogeneous sample, requiring *in vitro* enzymatic ubiquitination to be scaled up and combined with a purification protocol. Since the yields of the enzymatic ligation are often not sufficiently high and in some cases the E2 and E3 enzymes responsible for the reaction are not identified, researchers have developed several chemical (non-enzymatic) approaches that allow production of ubiquitinated proteins. Recently, a few examples of enzymatic ligation for biochemical/structural purposes have been published.

**Figure 1:**
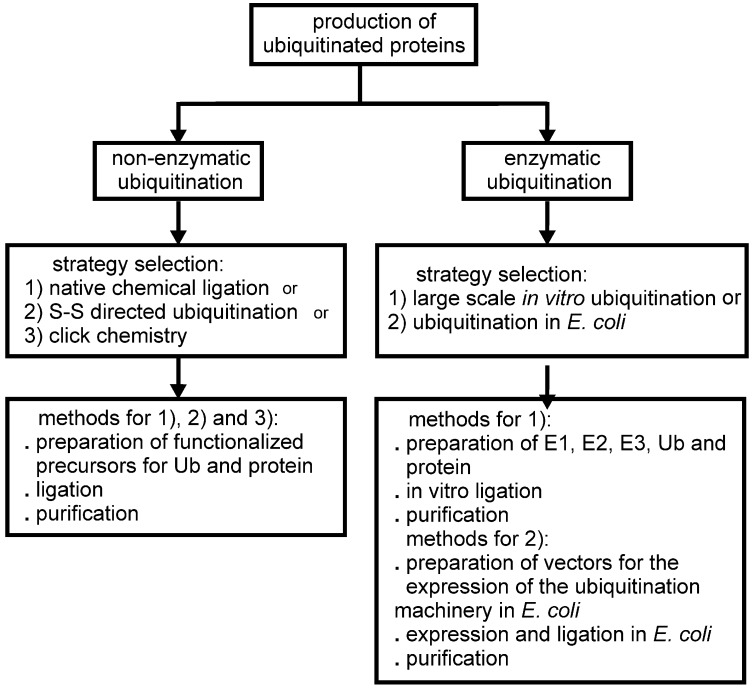
Schematic flow chart of the possible approaches developed so far for the production of mono-ubiquitinated proteins for biochemical and structural studies.

In this review, we discuss the different strategies applied for the production of ubiquitinated proteins listing the few examples so far available in the literature. We divide the different approaches to protein ubiquitination for biochemical/structural studies into two groups: (i) non-enzymatic (chemical) and (ii) enzymatic ubiquitination (Figure 1). Non-enzymatic methods consist of the introduction of reactive groups both onto the target protein at the position wanted for the covalent modification and onto the C-terminus of Ub. Different approaches have been developed. Enzymatic strategies, on the other hand, exploit the action of the enzymatic cascade of enzymes (E1, E2 and E3) that are responsible for protein ubiquitination *in vivo*. Several E2, E3 and DUBs have been linked to Ub at the reactive catalytic cysteine via a thioester bond using Ub aldheyde or Ub vinyl sulphone, and the structures of the products have been solved by X-ray crystallography (some examples are described in [4,5,6,7,8]). Also, Ub was attached enzymatically to the catalytic site of the E2 UbcH5 by mutating the catalytic C85 into a lysine [9]. The purpose of these studies is not strictly directed towards the investigation of the effects of protein ubiquitination on a lysine residue, but to entrap enzymatic reaction intermediates, so we do not include such examples in this review.

**Figure 2:**
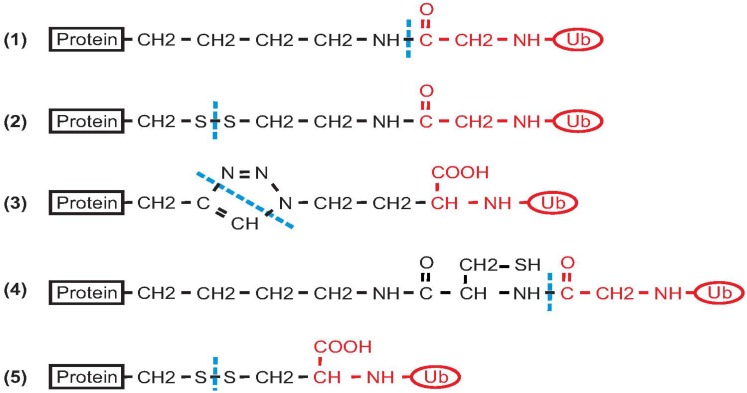
Summary of the different types of covalent bonds that result from different approaches for protein ubiquitination. (1) Native isopeptide bond; (2) Product of disulphide directed ligation; (3) Product of azide-alkyne cycloaddition; (4) Product of ligation using a genetically encoded d-cysteine-ε-lysine; (5) Simple disulphide ligation (protein and Ub precursors are functionalized by introduction of two cysteine residues). Black indicates the protein and the reactive linker. The Ub moiety is shown in red. A dashed blue line indicates the site(s) of covalent bond formation between the protein and Ub.

Most of the effort directed at producing ubiquitinated proteins for biochemical and structural studies has been focused on mono-ubiquitination. Ideally, the sample should have a native isopeptide bond between G76 of Ub and the specific lysine of the target protein. In some approaches to chemical ligation, however, the isopeptide bond is mimicked by other types of linkages of comparable length (Figure 2). On the other hand, enzymatic driven strategies have the advantage of producing native isopeptide bonds. Two recent papers reported synthetic methods for the production of poly-ubiquitinated proteins [10,11]. The reader is referred to the following reviews and papers for methods of production of isolated poly-Ub chains [12,13,14,15,16,17].

Table 1 summarizes all the examples of proteins for which successful mono-ubiquitination has been performed. We apologize to colleagues whose contribution we have unintentionally omitted.

**Table 1:** List of the mono-ubiquitinated proteins produced in different studies for biochemical or structural investigation. In the columns, from left to right: name of the protein; reference to the bibliography with classification as non-enzymatic or enzymatic approach; type of connection between Ub and the linked protein; buffer conditions that could be critical for the extension of the method to unstable proteins; stability to treatment with reducing agents; availability of structural information obtained from the mono-ubiquitinated sample, either using X-ray crystallography or NMR. When explicitly indicated by the Protein Data Base (PDB) code, the structural coordinates are available.

Protein Name	Method: Non-Enzymatic	Method: Enzymatic	Native Isopeptide Bond ^1^	Destabilizing Conditions	Stable to Reducing Agents	X-ray or NMR
Histone H2B	McGinty *et al.* [18]		yes (1)	6 M guanidine hydrochloride	yes	no
	Chatterjee *et al.* [19]		no (2)	6 M guanidine hydrochloride	no	no
PCNA	Chen *et al.* [20]		no (2)	no	no	no
	Freudenthal *et al.* [21]		no (no covalent bond)	no	yes	X-ray 3L10, 3L0W
	Eger *et al.* [22]		no (3)	no	yes	no
		Zhang *et al.* [23]	yes (1)	no	yes	X-ray 3TBL
α-synuclein	Hejjaoui *et al.* [24]		yes (1)	6 M guanidine hydrochloride	yes	no
	Meier *et al.* [25]		no (2)	6 M guanidine hydrochloride	no	no
calmodulin	Li *et al.* [26]		no (4)	no	yes	no
SUMO	Virdee *et al.* [27]		yes (1)	no	yes	no
Ras	Baker *et al.* [28]		no (5)	no	no	NMR
Josephin		Faggiano *et al.* [29]	yes (1)	no	yes	NMR
Rpn10		Keren-Kaplan *et al.* [30]	yes (1)	no	yes	no
Vps9		Keren-Kaplan *et al.* [30]	yes (1)	no	yes	no

^1^ The number in parenthesis refers to the numbering of the chemical formula shown in Figure 2.

## 2. Non-Enzymatic Methods

The synthetic methods proposed for the production of poly-Ub chains and mono-ubiquitinated proteins for structural investigation have been reviewed before [13,14,15]. Here, we only summarize the examples available in the literature that report on the chemical synthesis of ubiquitinated proteins. For each protein, we describe the rationale underlying the chemical ligation strategy, the experiments performed on the ubiquitinated product and discuss the advantages and the limits of the method.

### 2.1. Histone H2B

**Figure 3:**
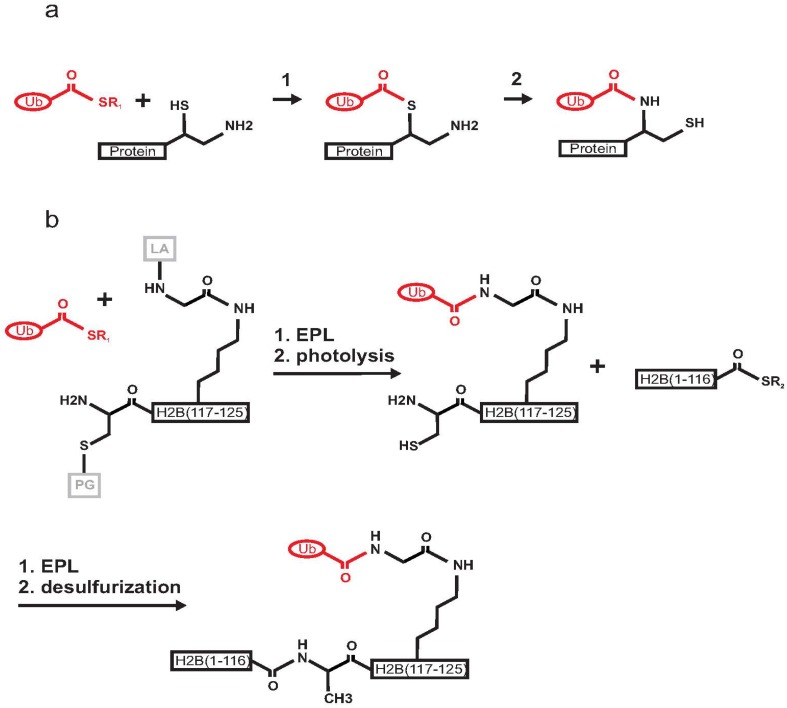
Schematic mechanism of expressed protein ligation (EPL) between Ub C-terminal-α-thioester and a protein having a 1,2-aminothiol group. (**a**) Reaction 1: transthioesterification step; Reaction 2: S-to-N acyl shift resulting in the formation of a native isopeptide bond. (**b**) Scheme of the semisynthesis of mono-ubiquitinated histone H2B. Ub (1–75) is in red, the two fragments forming H2B are in black, the cysteine protective group (PG) and the ligation auxiliary (LA) are in grey. The recombinant Ub C-terminal-α-thioester was ligated by EPL with an auxiliary linked synthetic peptide H2B (117–125). Photolysis allowed the removal of the ligation auxiliary and of the cysteine protective group. A second EPL step led to the ligation of recombinant H2B (1–116). Finally, mono-ubiquitinated H2B is formed by cysteine desulphurization.

Histone ubiquitination plays a crucial role in gene transcription and DNA damage response [31,32]. The first chemical semisynthesis of a mono-ubiquitinated protein was reported in 2008 by Muir and co-workers for histone H2B [18,33]. The authors utilized expressed protein ligation (EPL), a technique derived from native chemical ligation (NCL) [34] (Figure 3a). The strategy for the ubiquitination of histone H2B exploited the use of a photo-cleavable ligation auxiliary containing an N-terminal cysteine moiety that reacts in an EPL reaction with the Ub C-terminal α-thioester. Three polypeptide building blocks were prepared. In brief, the Ub (1–75) C-terminal α-thioester generated by intein chemistry was ligated to a synthetic peptide corresponding to residues 117–125 of H2B (Figure 3b). This step was followed by photolysis to remove an o-nitrobenzyl protecting group and the ligation auxiliary. The product was then ligated to the α-thioester of recombinant H2B (residues 1–116). A final desulphurization step was required to form the final product, *i.e.*, H2B mono-ubiquitinated via a native isopeptide bond on K120. The product was incorporated into nucleosomes. Biochemical studies in the presence of the methyltransferase hDot1L demonstrated that ubiquitination directly activates methylation of H3 on K4 and K79 through a yet uncharacterized allosteric mechanism [18,35]. This approach took advantage of the absence of native cysteines in both H2B and Ub. Another advantage was that the site of *in vivo* ubiquitination for H2B (K120) is not in the middle of the sequence but close to the C-terminus of the protein, thus making the chemical synthesis of the peptide H2B (117–125) feasible. The main disadvantage of this approach was the challenging multi-step synthesis, which might be a limitation if scaling-up were required for structural purposes. Nevertheless, the method represented an innovative and elegant way to produce a native isopeptide bond between a target protein and Ub.

A simplified approach based on disulphide chemistry was proposed by the same group in 2010 [19,36]. An Ub-intein fusion protein was used to introduce a C-terminal amino ethanethiol group (Ub-SH). A K120C mutation was introduced into H2B, and the cysteine was activated for asymmetrical disulphide ligation by 2-thio-(5-nitropyridine). A disulphide-linked (S-S) analogue of mono-ubiquitinated H2B was formed under denaturing conditions. This product was shown to have a biochemical activity comparable to that of mono-ubiquitinated H2B obtained by EPL. While the disulphide-directed approach could in principle be more flexible than EPL, it requires a target protein with no native cysteine residues or, alternatively, the mutation of the native cysteines to serines. The instability of the S-S conjugate under reducing buffer conditions could also represent a limitation for this method.

### 2.2. PCNA

The protein PCNA is involved in DNA replication and repair, cell cycle control and chromatin remodelling [37]. During DNA damage response, K164 of PCNA is both ubiquitinated and sumoylated [38]. A protocol for the chemical ubiquitination of PCNA was first proposed by Zhuang and co-workers [20]. It consists of a direct disulphide bond formation. This approach is similar to that proposed for H2B and the two papers were published in the same journal issue [19,20]. The ligation of either Ub or SUMO required the use of a PCNA mutant with a unique cysteine introduced to replace K164 and in which four native cysteines were mutated to serines. Ub-SH obtained by intein chemistry was activated by the formation of a reactive asymmetric disulphide with 5,5'-dithiobis(2-nitrobenzoic acid) (DTNB). The authors produced 4.5 mg of mono-ubiquitinated PCNA with a final yield of 80%. The product is stable for 5 min at 37 °C in a 0.5 mM solution of reducing agents (dl-dithiothreitol (DTT) and gluthatione). However, 5 mM DTT at 25 °C cleaves the disulphide bond almost completely within 10 min.

In the same year, another group [21] published a method to produce mono-ubiquitinated PCNA by splitting the protein into two polypeptides that self-assemble by non-covalent interactions. The crystal structure of split mono-ubiquitinated PCNA was solved to a resolution of 2.8 Å and deposited in the Protein Data Bank (PDB codes 3L10 and 3L0W). The relative orientation of Ub and PCNA in the mono-ubiquitinated protein was described. Based on these results, the authors suggested that the role of ubiquitination could consist of the formation of a new binding surface to facilitate the recruitment of non-classical DNA polymerases.

One year later, Marx and co-workers produced mono-ubiquitinated PCNA using click chemistry [22]. Unnatural amino acids were introduced so that an azide and an alkyne group were introduced on Ub and PCNA, respectively. The two proteins were then covalently linked by a Cu(I)-catalyzed azide-alkyne cycloaddition. In the product, the isopeptide bond is mimicked by a stable triazole linkage. The authors showed that the synthetic mono-ubiquitinated PCNA is able to stimulate DNA synthesis by DNA polymerase δ, and that the affinity of DNA polymerase η for PCNA increases upon ubiquitination.

The first record of the crystal structure of covalently mono-ubiquitinated PCNA was deposited in PDB in 2012 [23]. This protein, however, was produced by enzymatic ubiquitination (see Section 2.1. for details), suggesting that this method was a more suitable strategy for large-scale preparation.

### 2.3. α-Synuclein

Parkinson’s disease is associated with the formation of toxic aggregates of the protein α-synuclein, which is found to be post-translationally modified by Ub at several different lysine residues [39]. To investigate the effect of ubiquitination of a specific lysine, semi-synthetic mono-ubiquitinated α-synuclein was produced [24]. The semi-synthesis of K6 mono-ubiquitinated α-synuclein was achieved by EPL. A recombinant fragment of α-synuclein (residues 19–140) bearing an N-terminal cysteine residue was ligated to a synthetic peptide thioester (residues 1–18) with a δ-mercapto lysine replacing K6. This approach was feasible because α-synuclein lacks native cysteine residues. After the first ligation step, C19 was converted into alanine and an intein-derived Ub thioester was simultaneously linked to α-synuclein (1–140) through the δ-mercapto lysine functional group. Finally, desulphurization conditions were applied to form the native mono-ubiquitinated protein. The product was characterized by circular dichroism (CD), thioflavin T (ThT) binding fluorescence assays and transmission electron microscopy (TEM).

Disulphide-directed ubiquitination of α-synuclein was used to generate nine site-specifically Ub modified derivatives and demonstrates that different ubiquitination sites have differential effects on the aggregation of the protein [25]. Ub was expressed in *E. coli* as an intein-fusion protein. Intein-mediated thiolysis with cysteamine and subsequent reaction with DTNP generated an activated Ub disulphide. Recombinant α-synuclein cysteine point-mutants (having each lysine individually mutated to a cysteine) were then reacted with the activated Ub to generate the corresponding disulphide-directed mono-ubiquitinated derivates. The same authors demonstrated that site-specific effects of mono-ubiquitination support different levels of α-synuclein degradation using an *in vitro* proteasome turnover assay [40].

### 2.4. Calmodulin

Another approach for producing ubiquitinated proteins is the incorporation of an unnatural aminoacid to introduce a ligation handle into a recombinant protein. This strategy was exploited by Chan and co-workers for the production of mono-ubiquitinated calmodulin [26]. Ubiquitination of calmodulin at lysine 21 modulates the regulatory properties of the protein and thus affects calcium signalling in cells [41,42]. The authors introduced a genetically encoded pyrrolysine analogue (D-cysteine-ε-lysine) at position 21 of calmodulin using a pyrrolysyl-tRNA synthetase/tRNA pair from *M. barkeri*. The modified calmodulin was then ligated through NCL with Ub C-terminal-α-thioester obtained by intein chemistry. Mono-ubiquitinated calmodulin was used to explore the effect of ubiquitination on calmodulin-mediated regulation of phosphorylase kinase and protein phosphatase 2B. The main challenge of this approach is the incorporation of an unnatural aminoacid in the target protein. In this example, the linker between the protein and ubiquitin is slightly longer than a proper native isopeptide connection (Figure 2).

### 2.5. SUMO

**Figure 4:**
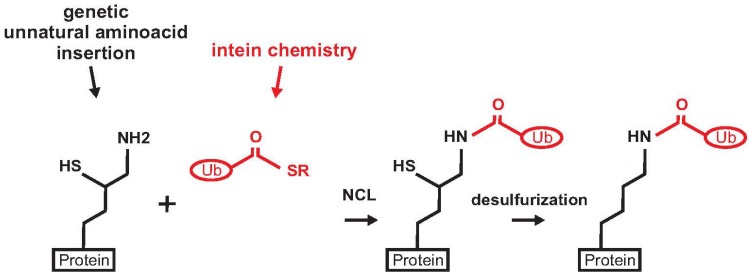
Scheme of the strategy for the preparation of mono-ubiquitinated SUMO. The protein (SUMO) is in black, Ub C-terminal α-thioester is depicted in red.

A different strategy for protein ubiquitination exploiting the genetic encoding of an unnatural aminoacid was proposed by Chin and co-workers [27]. This approach has the advantage of producing a native peptide bond without using denaturing buffer conditions. The method is an evolution of the genetically encoded orthogonal protection and activated ligation (GOPAL), developed by the same group for the production of Ub chains. This method requires multiple protection and deprotection steps and refolding of the substrate protein [43]. The SUMO protein was chosen as a model to set up a strategy for traceless and site-specific protein ubiquitination. A δ-mercapto-lysine group was genetically introduced at position 11 of recombinant SUMO (Figure 4). The protein was then ligated to the Ub C-terminal α-thioester. A final desulphurization step produced the native mono-ubiquitinated SUMO.

### 2.6. Ras

Ras is the most common oncogene in human cancers, and its protein product performs a crucial role in cell growth, differentiation and apoptosis [44]. Mono-ubiquitination of Ras at K147 has been shown to promote tumorigenesis. The structural effects of Ras ubiquitination were investigated using disulphide-directed ligation [28]. The isopeptide bond was mimicked by a simple strategy: a disulphide bond was formed between the C-terminus of a UbG76C mutant and RasK147C. The solvent-accessible C118 of Ras was mutated to serine to prevent unwanted modifications, while the other two cysteines in the construct used (spanning residues 1–166) did not react, because these are not exposed on the surface. NMR characterization was carried out on mono-ubiquitinated Ras, which demonstrated that mono-ubiquitination renders the protein resistant to GAP-mediated regulation. The same authors also investigated, via NMR, the effect of ubiquitination at two different sites (K117 and K147) [45]. Although the linker between the protein and Ub is shorter than in a native isopeptide bond (Figure 2), this method allowed the authors to obtain important functional data on Ras activation.

### 2.7. Production of Poly-Ubiquitinated Proteins

The same research group that published the first structure of mono-ubiquitinated α-synuclein implemented a strategy for the production of the protein linked to di- and tetra-Ub chains. It employs isopeptide chemical ligation (ICL) [10]. Lys48-linked di- or tetra-Ub chains were incorporated into the side chain of K12 of α-synuclein, having δ-mercapto lysine at the desired lysine residue. The α-synuclein moiety was prepared exploiting an approach similar to that described above for mono-ubiquitination at K6; a chemically synthesized α-synuclein thioester peptide (residues 1–29) was linked to a recombinant α-synuclein fragment (30–140) harbouring an N-terminal cysteine. Two different fragments of di-Ub thioester building blocks were attached to the protein in two sequential ligation steps, forming the di- and tetra-ubiquitinated products. The protocol requires two reaction steps at pH 4 with methoxyamine to unmask the reactive δ-mercaptolysine groups on α-synuclein (first step) and at position 48 of the distal Ub in the attached di-Ub moiety (second step), followed by a final desulphurization step. The poly-ubiquitinated α-synuclein products obtained were used to investigate the effect of the Ub chain length on the proteosomal degradation of the protein.

A recent paper from Brik’s group reported another novel strategy for the chemical synthesis of poly-ubiquitinated proteins [11]. This approach is based on the ligation of a cysteine in a target protein to a synthetically produced poly-Ub chain that contains a designed electrophile derived from the C-terminal hydrazide group of Ub-NHNH2 at the proximal end. The strategy does not allow formation of a native isopeptide bond, but it represents an alternative to the introduction of unnatural amino acids, *e.g.,* δ-mercaptolysine (as in [10]). α-Globin was used as a reference protein to assess poly-ubiquitination. The protocol requires the synthesis of mono-Ub building blocks, the assembly of the Ub chain, and the conjugation of the Ub chain to α-globin. After assembly of the Ub chain, tetra-Ub-NHNH2 was ligated to C104 of α-globin via formation of a disulphide bond. In the same paper, the authors also propose alternative conjugation strategies leading to the formation of a more stable thioether or maleimide linkage between α-globin and di-Ub. The advantage of these groups over a disulphide bond is that they are resistant to reducing conditions, while the disulphide conjugates are quickly cleaved by the reducing agent in the buffer. This advantage, for instance, allowed the use of the DUB USP2 for enzymatic cleavage of the Ub-Ub bond of di-ubiquitinated α-globin.

## 3. Enzymatic Methods

Along with the chemical strategies described above, a series of enzymatic directed protocols have been developed recently to produce ubiquitinated samples suitable for biochemical and structural studies. The idea underlying these strategies is that the formation of a native isopeptide bond between Ub and a given protein can be catalyzed by the coordinated action of E1, E2 and E3 enzymes. This approach can potentially lead to the production of large amounts of ubiquitinated samples, conjugated by native isopeptide bonds. The main limitation is that E2 and E3 enzymes, able to perform the ubiquitination of a specific protein on the target lysine residue, must be known. Also, multi-ubiquitination on different lysine residues might occur (unless lysine to arginine protein mutants are used), and the mono-ubiquitinated product has to be purified from possible poly-ubiquitinated and unreacted species.

### 3.1. PCNA

**Figure 5:**
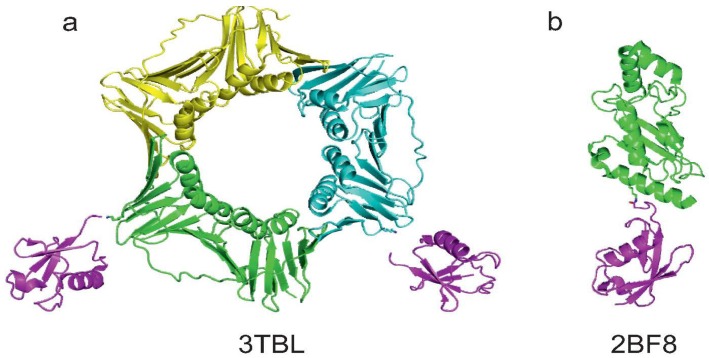
Summary of the currently available structures of ubiquitinated proteins. (**a**) Structure of mono-ubiquitinated PCNA (PDB code 3TBL). Note that the last GG moieties on the two Ub molecules do not appear in the PDB structure. K164 of two of the three monomers of the PCNA trimer are showed as sticks; (**b**) Structure of sumoylated E2-25K (PDB code 2BF8). K14 of E2-25K and G97 of SUMO are reported as sticks to visualise the native isopeptide bond.

The first crystal structure deposited in PDB of a protein mono-ubiquitinated on a lysine residue known to be conjugated with Ub *in vivo* is PCNA [23] (code 3TBL). The protein was produced by *in vitro* ubiquitination, using the same protocol reported in a previous paper [46]. Ub, E1, UbcH5c (E2) and RNF8 (E3) were mixed with PCNA in the presence of ATP and MgCl_2_. The resulting protein was mono-ubiquitinated specifically on K164, despite the fact that other lysines are present in the sequence. The authors managed to crystallize mono-ubiquitinated PCNA and solve the X-ray structure of the covalent complex (Figure 5a). This result suggests that *in vitro* enzymatic ubiquitination is in principle a suitable approach to obtain a sample that is mono-ubiquitinated on a specific lysine residue.

### 3.2. Josephin

We proposed a similar enzymatic approach for the production of mono-ubiquitinated Josephin, the catalytic domain of ataxin-3 [29]. This protein is a DUB that preferentially cleaves poly-ubiquitinated chains and, when mutated, is involved in the neurodegenerative Machado-Joseph disease [47,48,49,50]. *In vivo*, Josephin is ubiquitinated on K117, and this modification increases the enzymatic activity of the protein [51,52,53]. In order to understand the molecular mechanism underlying the activation, we prepared mono-ubiquitinated Josephin using large scale *in vitro* ubiquitination. Among the other E2/E3 enzymes able to ubiquitinate ataxin-3, we chose UbcH5a and CHIP as they were shown to perform ubiquitination on K117. E1 from insect cells, UbcH5a (E2) and CHIP (E3) from *E. coli* expression were prepared to catalyze the reaction in the presence of ATP and MgCl_2_. We used a Josephin mutant in which all lysines except for K117 were mutated to arginines to obtain a homogeneous sample suitable for structural characterization. As a preliminary requirement, we verified that these conservative mutations do not influence protein structure and activity [29]. The enzymes catalyzed the formation of mono-ubiquitinated Josephin, but also of poly-ubiquitinated products. We therefore set up a protocol for the purification of mono-ubiquitinated Josephin, consisting of a single step of anion exchange chromatography. This approach allowed us to obtain milligrams of mono-ubiquitinated protein with a native isopeptide bond. A clear advantage of using an enzymatic approach was that it made it possible to use differential isotopic labelling schemes; Josephin and Ub in the mono-ubiquitinated product could be alternatively labelled with ^15^N and ^13^C isotopes during the expression in *E. coli*, allowing a structural characterization of the covalent complex by NMR.

### 3.3. Rpn10 and Vps9

An alternative enzymatic strategy was proposed in 2012 [30]. The enzymatic cascade necessary for ubiquitination of proteins, such as Rpn10 and Vps9, was reconstituted in *E. coli*. Two expression vectors were used: one containing Ub, E1 and the selected E2, the second encoding the selected substrate for ubiquitination plus the required E3 enzyme. Both Ub and the protein substrate were affinity tagged, enabling the ubiquitinated product to be purified to homogeneity in quantities sufficient for structural studies. The yield of ubiquitinated proteins was 0.5–1 mg of purified product per litre of culture. In the case of Rpn10, the reconstituted system (expressing UbcH5b and Rsp5 as E2 and E3, respectively) succeeded in ubiquitinating only the lysine residue that is modified *in vivo*, *i.e.*, K84. The authors demonstrated that the enzymatic cascade reconstituted in bacteria conserves its specificity and fidelity. Vps9 was ubiquitinated with the same system via co-expression with Ubc4/5 as E2 and Rsp5 as E3. Several lysines of Vsp9 were modified. This work allowed the authors to perform Förster resonance energy transfer (FRET) experiments on ubiquitinated Vps9 and investigate the effect of ubiquitination on the activity of the protein.

### 3.4. Sumoylation of E2-25K

Although E2-25K is not modified with Ub, but with SUMO, we briefly report the work on sumoylation of this protein as an example of modification via enzymatic ligation that permitted the solution of the X-ray structure of the covalent complex [54]. The Ub conjugating enzyme E2-25K (residues 1–155) was covalently linked to SUMO1 using E1 and Ubc9 in the presence of ATP and MgCl_2_. Sumoylated E2-25K was then purified by size-exclusion and anion-exchange chromatography. The product was crystallized and the coordinates of the complex were deposited in PDB with the code 2BF8 (Figure 5b).

## 4. Conclusions

We have presented here several examples of enzymatic and non-enzymatic approaches that permit the synthesis of ubiquitinated proteins. Great effort was invested in recent years to produce Ub chains with different connectivities and/or ubiquitinated proteins. When comparing non-enzymatic and enzymatic approaches, it is clear that both methods have advantages and disadvantages.

Non-enzymatic ligation permits chemically directed ligation on a particular amino acid of the sequence. However, not all chemical strategies that have been conceived lead to the formation of a native isopeptide bond; in several examples discussed here, the isopeptide bond is mimicked by a disulphide or a triazole group. It can thus be argued that the isopeptidemimic might not always be a good model. Also, if disulphide chemistry is used, the mono-ubiquitinated protein formed is unstable in reducing buffers. Another limitation of the non-enzymatic methods is that most chemical ligation approaches require a laborious multi-step synthesis that considerably reduces the final yield.

The synthesis of ubiquitinated proteins also induces a general complication that arises from the potential instability of the target protein to drastic conditions of pH, solvent and reactants (e.g., denaturing buffer containing 6 M guanidine hydrochloride used for the synthesis of histone H2B or α-synuclein). In general, non-enzymatic strategies developed for the synthesis of poly-Ub chains are not necessarily suitable for the production of ubiquitinated proteins. In fact, Ub is a robust protein that is stable even under extreme pH conditions and easily able to refold reversibly after denaturation. This is unfortunately not true for most protein targets of ubiquitination. Some approaches for the synthesis of Ub chains, for example, require refolding after ligation, which might be a limiting step for most proteins. Others involve reaction steps at very low pH (e.g., pH 3), a condition that would induce precipitation and denaturation of most proteins.

In comparison, enzymatic ligation is a more “natural” approach, which always leads to the formation of a native isopeptide bond in buffer conditions that are in most cases favourable to protein stability (neutral pH). In some cases, however, there is the complication that poly-ubiquitinated products may form, thus requiring additional purification steps to obtain a homogeneous sample. The reaction might also not be solely directed towards the (physiologically relevant) expected lysine residue(s), therefore requiring preceding mutations of the other lysines to arginines. If such mutations are introduced, it is necessary to check experimentally that the protein structure and function are not affected. Another disadvantage is that these methods require the production of E1, E2 and E3 enzymes in large quantities. But overall, the main limitation of these methods is that the identity of the specific E2 and E3 enzymes needed to catalyze the reaction must be known. This problem can, in principle, be surmounted, as there are examples in the literature of E3-independent ubiquitination or sumoylation [54,55]. Screenings to identify an efficient E2 enzyme, eventually associated to mutations of lysines to arginines, could thus be a productive approach to obtain ubiquitination of a target protein even without specific pre-knowledge. Much more effort will be needed in the future to design and standardize general methodologies to obtain ubiquitinated proteins.
